# Decibel Decisions: The Concept of Intracranial Aneurysm Surgery With a Decibel Meter on Two Surgical Cases

**DOI:** 10.7759/cureus.48993

**Published:** 2023-11-18

**Authors:** Manuel de Jesus Encarnacion Ramirez, Feres Chaddad-Neto, Nicola Montemurro, Issael Jesus Ramirez Pena, Andreina Rosario Rosario, Carlos Catillo-Rangel, Gilberto González López, Juan J Cardona, Alvaro Campero, Matias Baldoncini

**Affiliations:** 1 Department of Neurological Surgery, Peoples’ Friendship University of Russia, Moscow, RUS; 2 Department of Neurological Surgery, Universidade Federal de São Paulo, São Paulo, BRA; 3 Department of Neurosurgery, Azienda Ospedaliero Universitaria Pisana, Pisa, ITA; 4 Department of Neurooncology, Royal Melbourne Hospital, Melbourne, AUS; 5 Department of Neurosurgery, Autonomous University of Santo Domingo, Santo Domingo, DOM; 6 Neurosurgery, Servicio of the 1ro de Octubre Hospital of the Instituto de Seguridad y Servicios Sociales de los Trabajadores del Estado, México City, MEX; 7 Department of Dubai Health Authority, Ministry of Health and Prevention, Dubai, ARE; 8 Department of Neurosurgery, Tulane University School of Medicine, New Orleans, USA; 9 Department of Neurological Surgery, Hospital Padilla, Tucumán, ARG; 10 Department of Neurosurgery, University of Buenos Aires, Buenos Aires, ARG

**Keywords:** cerebral aneurysm surgery, microsurgical aneurysm clipping, micro-doppler, neurovascular surgery, decibel meter mobile

## Abstract

The present cases were used to investigate the reliability of the intraoperative decibel meter as an objective method of clipping efficiency in cerebral aneurysm surgery and to assess the impact of this method on the surgical procedure itself. Different methodologies have been developed and applied to directly or indirectly evaluate the placement of a clip, for example, intraoperative digital subtraction angiography (DSA), intraoperative micro-Doppler ultrasonography, and, more recently, indocyanine green (ICG). We included two patients with a previously non-treated unruptured brain aneurysm. In both patients, intraoperative micro-Doppler was used in combination with a decibel meter app. Here, we present the cases of two patients. In patient one, the pre-clipping average sound level/equivalent continuous sound pressure level (Avg/Leq) was 96.7 dB, while the post-clipping Avg/Leq was 94.4 dB, indicating a reduction in sound level after clipping. Similarly, the pre-clipping time-weighted average noise level (TWA) was 1.2%, while the post-clipping TWA was 0.5%, indicating a decrease in exposure dose after clipping. In patient two, the average sound level for the post-clipping measurement (94.2 dB) was higher than the pre-clipping measurement (93.5 dB), but the difference was not statistically significant. These cases indicate the potential for using sound measurements as a reliable indicator of adequate aneurysm occlusion during clipping procedures. Further research is needed to confirm these findings.

## Introduction

The outcome of surgical treatment of cerebral aneurysms may be severely impaired by local cerebral ischemia or by infarction resulting from the inadvertent occlusion of an adjacent vessel. Incomplete aneurysm occlusion, on the other hand, increases the risk of hemorrhage [1]. In many cases, intraoperative evaluation of post-clipping status is difficult. Different methodologies have been developed and applied to directly or indirectly evaluate the placement of a clip, for example, intraoperative digital subtraction angiography (DSA), intraoperative micro-Doppler (MD) ultrasonography, and, more recently, indocyanine green (ICG). However, DSA is associated with high costs and requires highly trained personnel, which can be difficult to obtain [2].

It is crucial that both aneurysm occlusion and inadvertent clipping of neighboring vessels are avoided during the surgical procedure. Doppler ultrasonography was the first technique used for the assessment of cerebral hemodynamics in extracranial vessels. Aaslid et al. [3] modified this technique for the transcranial investigation of cerebral vessels. Technical progress made it possible to reduce the size of the ultrasound probe by increasing the ultrasound frequency. Martin et al. [4], using intraoperative angiography, showed that 8.8% of the treated aneurysms were inadequately clipped despite visual verification of each of the vessels. Hence, confirming the blood flows of the different afferent and efferent vessels and the dome of the aneurysm during pre and post-clipping plays a fundamental role in avoiding ischemic events and improving patient outcomes [4]. The use of vascular MD in brain aneurysm surgery is well documented, demonstrating its usefulness as a guide for adequate clip positioning. This technique is non-invasive, cheap, and fast and allows the neurosurgeon to qualitatively evaluate the speed of blood in the artery and the absence of flow in the dome of the aneurysm after clipping [5].

We theorize that the use of MD with concomitant measuring of sound can provide an accurate intraoperative evaluation of clip position. This technique is fast, cheap, and easy to use; further, evidence suggests that it should be standard in clipping of cerebral aneurysms. By taking advantage of the widespread cell phone apps, the surgeon can have significant live information about the changes in the decibels (dB) at the clipped complex during different stages of the surgery.

In 1842, the Austrian physicist and mathematician Christian Doppler formulated the principle of the Doppler effect to try to explain the color of stars [6]. In 1982, Aaslid began to use transcranial Doppler in the study of patients with cerebrovascular diseases. This principle of Doppler ultrasound establishes the relationship between the speed of a moving object and the change in emitted frequency, the speed of the object, and the cosine of the angle of incidence, according to the following expression: f = 2 v f0 cosq / c, where f0 is the emitted frequency, v is the speed of the object, q is the angle of incidence and the speed of propagation of the wave in the medium [7]. Technological progress has reduced the probes and increased ultrasonographic frequency, making possible the direct study of cerebral vessels using MD [1,8,9].

This method has the potential to provide real-time feedback to the surgeon, which can help optimize the surgical procedure and improve patient outcomes. However, the reliability and impact of this method on the surgical procedure itself remain unclear.

This study was performed to investigate the reliability and practicability of intraoperative MD combined with a dB meter application during cerebral aneurysm surgery and to assess the influence of this method on the surgical procedure. The findings of this study can have significant implications for the optimization of cerebral aneurysm surgery and improve patient outcomes.

## Case presentation

We present the cases of two patients with a previously non-treated unruptured brain aneurysm. Both patients undergoing surgical treatment for cerebral aneurysms were recruited for this study. Patient one is a 57-year-old female with a 5.6 mm aneurysm located in the right middle cerebral artery (segment M1). Patient two is a 63-year-old male with a 3.8 mm aneurysm located in the left middle cerebral artery (segment M3). In all of these procedures, intraoperative MD was used in combination with a dB meter app. MRI, MR angiography (MRA), and time-resolved three-dimensional MRA and DSA were performed preoperatively in both patients to determine the surgical route. Both patients provided written informed consent to participate in the study.

The surgical procedure was performed under general anesthesia. Intraoperative MD ultrasonography was performed before and after aneurysm clipping to evaluate blood flow in the arteries and aneurysm dome. A dB meter was also used during the clipping procedure to measure sound levels in the clipped complex. The dB meter was positioned adjacent to the aneurysm dome and measurements were taken at different stages of the clipping procedure, including before, during, and after clipping. The sound measurements were recorded in dB and stored for later analysis.

Ultrasound examination of the afferent and efferent vessels was performed following the surgical exposure. We used the Mizuho Vascular Doppler Systems frequency 20 MHz, which provided audio blood flow detection at different depths of penetration before and after clip placement. A direct probe-to-vessel contact was established using forceps for convenient handling and reliable measurement. According to our Doppler protocol, the angle between the blood vessel and the probe was set at 60 degrees, and a sterile saline solution was used as the contact medium.

The data collected from the dB meter were analyzed to evaluate the effectiveness of this method in assessing clipping efficiency. The dB measurements were compared to the results of the MD ultrasonography to determine if sound levels can be used as a reliable indicator of adequate aneurysm occlusion. Any discrepancies between the two methods were noted and analyzed. The impact of the use of the dB meter on the surgical procedure was also assessed by reviewing surgical notes and recordings. Statistical analysis was performed using the paired t-test to determine the significance of the observed changes. A p-value <0.05 was considered statistically significant. The study protocol was approved by the local bioethics committee board and was conducted in accordance with the principles of the Declaration of Helsinki. Patient confidentiality was maintained at all times and all data were kept confidential and anonymous. Both patients provided written informed consent to participate.

Patient one

The hypothesis that clipping reduces the average sound level (average sound level/equivalent continuous sound pressure level (Avg/Leq)) and exposure dose (time-weighted average noise level (TWA)) compared to pre-clipping levels is supported. The pre-clipping Avg/Leq was 96.7 dB, while the post-clipping Avg/Leq was 94.4 dB, indicating a reduction in the sound level after clipping. Similarly, the pre-clipping TWA was 1.2%, while the post-clipping TWA was 0.5%, (Table 1), indicating a decrease in exposure dose after clipping.

**Table 1:** Acoustic monitoring and statistical analysis for patient one’s aneurysm. Avg: average sound level; dB: decibel; Leq: equivalent continuous sound pressure level; TWA: time-weighted average noise levels

Measurement	Time (s)	Avg/Leq (dB)	Min (dB)	Max (dB)	Peak (dB)	TWA (dB)	Dose (%)	Projected doses (%)	P-value
Pre-clipping	23	96.7	68.8	100.4	104.1	65.6	1.2	1,476.1	N/A
Post-clipping	15	94.4	79.5	98.6	100.9	61.6	0.5	856.4	0.3

**Figure 1:**
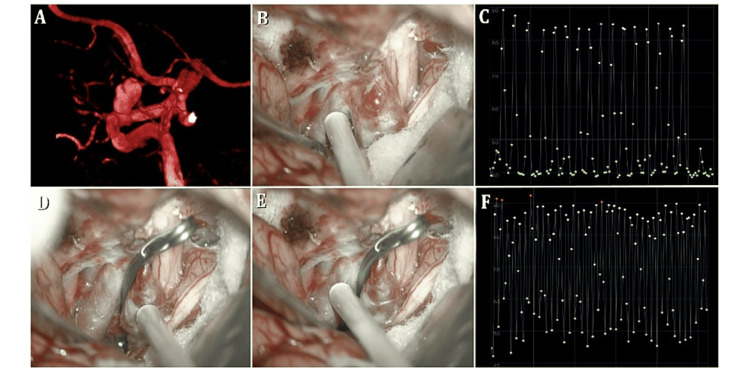
Patient one. (A) Ophthalmic carotid aneurysm on the left side. (B) The aneurysm is fully exposed after performing an anterior clinoidectomy. (C) Doppler decibelometry on the internal carotid in the post-aneurysm ophthalmic segment shows an average sound of 80 dB. (D) After clipping, we performed a Doppler on the aneurysm, which was negative. (E) We then placed the Doppler in the same location. (F) Doppler decibelometry shows the same sound intensity of 80 dB, confirming that the Doppler reading is exactly the same before and after clipping.

The statistical analysis performed using a two-tailed t-test with a significance level of 0.05 revealed a p-value of 0.03. These findings indicate that there is a statistically significant difference in average sound levels between the two conditions. The post-clipping condition exhibited a lower average sound level (Avg/Leq) compared to the pre-clipping condition. This suggests that the clipping process had a measurable impact on sound levels, and this difference was unlikely to have occurred by random chance alone (Figure 1).

Patient two

The hypothesis is that clipping reduces the average sound level (Avg/Leq) and exposure dose (TWA) compared to pre-clipping levels (Table 2). 

**Table 2:** Acoustic monitoring and statistical analysis for patient two’s aneurysm. Avg: average; db: decibel; Leq: equivalent continuous sound level; TWA: time-weighted average sound level

Measurement	Time (s)	Avg/Leq (dB)	Min (dB)	Max (dB)	Peak (dB)	TWA (dB)	Doses (%)	Projected doses (%)	P-value
Pre-clipping	20	93.5	88.3	96.9	100.7	61.9	0.5	704.6	N/A
Post-clipping	17	94.2	69.7	98.3	105.5	61.9	0.5	822.3	0.48

**Figure 2:**
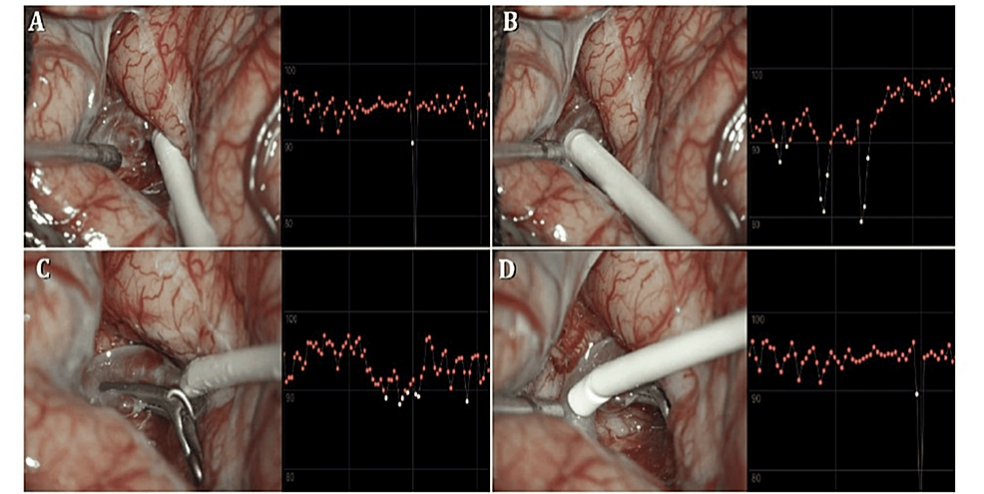
Patient two. (A) Exposes a Sylvian aneurysm at a left bifurcation. Here, the Doppler is placed on the superior or frontoparietal branch of the middle cerebral artery. The Doppler dB reading is shown on the right margin, highlighting that the superior branch has an intensity of 67 dB. (B) The inferior temporal branch averages at 63 dB. (C) After clipping, the intensity remains consistent at 67 dB. (D) Shows the average dB reading.

**Figure 3:**
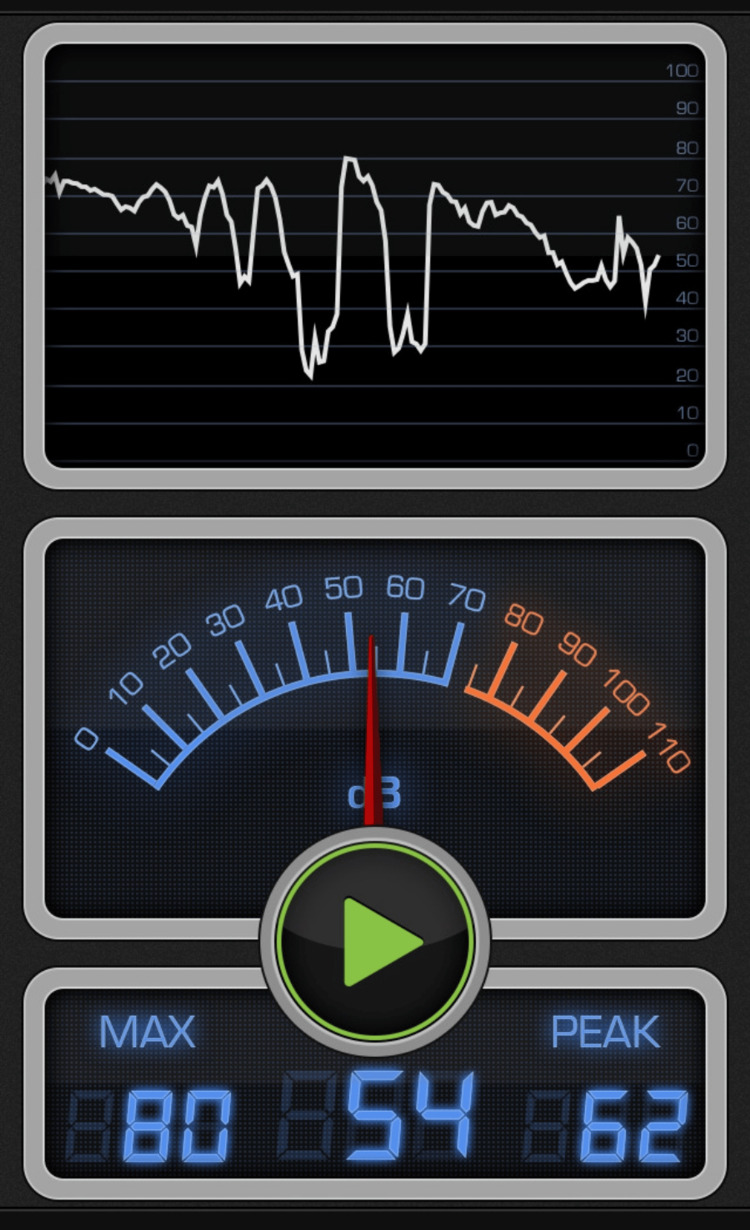
Decibel X. Decibel X is a tool that we used to measure sound intensity from our Android phone.

The hypothesis that clipping reduces the average sound level (Avg/Leq) and exposure dose (WAS) compared to pre-clipping levels can be supported. The average sound level for the post-clipping measurement (94.2 dB) was higher than the pre-clipping measurement (93.5 dB), but the difference was not statistically significant (p = 0.048). However, the TWA for the post-clipping measurement (61.9 dB) was the same as the pre-clipping measurement, while the projected dose was higher for the post-clipping measurement (822.3%) compared to the pre-clipping measurement (704.6%). Therefore, it appears that clipping may reduce exposure dose in some cases, but this finding should be interpreted with caution and further research is needed to confirm the results. As the p-value (0.48) was greater than the chosen significance level (0.05), we would fail to reject the null hypothesis. This suggests that there is not enough evidence to conclude that there is a significant difference in average sound levels (Avg/Leq) between the pre-clipping and post-clipping conditions (Figures 2, 3).

## Discussion

The novel approach of using a dB meter in conjunction with intraoperative MD presented an intriguing possibility for enhancing the assessment of cerebral aneurysm clipping efficiency. The results from the two cases analyzed in this study provide preliminary insights into the potential of this combined methodology. For patient one, results showed a statistically significant reduction in sound levels post-clipping. This decrease in sound level aligns with the theory that clipped aneurysms might lead to a change in blood flow dynamics, which, in turn, could potentially be detected as changes in sound levels by the dB meter. This lends credibility to the idea of using sound as an adjunctive tool in evaluating clipping efficiency. However, the findings for patient two introduce an element of caution. While there was a difference in dB readings before and after clipping, it was not statistically significant. The variability in results between the two patients suggests that individual anatomical differences, variations in aneurysm morphology, or even the relative size and location of the aneurysm might influence the sound levels detected by the dB meter. Furthermore, the lack of a statistically significant difference in patient two brings forth the question: How much of a change in sound is clinically relevant? The use of MD during surgery allows evaluation of the hemodynamics of cerebral vessels, enabling the study of proximal, distal, and intra-aneurysmal flow [10]. The MD recordings can be differentiated into the following three categories: normal signal, with regular laminar flow aspect of adjacent vessels; weak signal, with irregular appearance of the whirling flow of the aneurysmal sac; and pertinent to the total occlusion of the aneurysmal sac or a vessel adjacent (occlusion or stenosis) [11]. The prominence of MD in the field of neurosurgery has seen a noteworthy ascent, positioning itself as an indispensable asset for neurosurgeons worldwide. Its ability to provide real-time visualization of blood flow within cerebral vessels has not only bridged the gap between visualization and surgical precision but has also played a pivotal role in augmenting patient outcomes [12].

The fundamental challenge in these procedures is not just the occlusion of the aneurysm but also ensuring the patency of adjacent vessels. The landmark study by Siasios et al. [5] emphasized the unparalleled capability of MD in ensuring optimal surgical outcomes. It does so by providing a real-time confirmation of the absence of blood flow within the aneurysm post-clipping. This, coupled with its ability to verify the uninterrupted flow within neighboring arteries, is a testament to its indispensability in aneurysm surgeries [13] Moreover, in bypass procedures, the dynamics of the surgery revolve around graft patency and the restoration of blood flow. Morisawa et al. [14] provided a comprehensive insight into this, showcasing how MD stands as a beacon for surgeons, aiding in the confirmation of graft patency. Its role in verifying the restoration of flow post-surgery, thereby ensuring the graft’s functionality, cements its position as a quintessential tool in bypass surgeries [15,16].

Based on the data presented, it appears that clipping can reduce both the average sound level and exposure dose during surgical procedures for aneurysm occlusion. The results of patient one show a significant difference in both sound level and exposure dose between pre and post-clipping measurements, while the results of patient two suggest a potential reduction in exposure dose but not a statistically significant reduction in sound level. The advent of the dB meter in the neurosurgical field is a testament to the continuous evolution of medical technology and its inherent desire to improve patient outcomes. Intriguingly, despite its evident potential, as of now, there has been no extensive documentation or published articles detailing its application within the context of neurosurgery [15,16]. This gap in the literature underscores both an opportunity and a challenge for the medical community. The lack of established literature on the use of a dB meter in neurosurgery is puzzling, given its profound implications [15]. The tool, which measures sound in terms of dBs, offers a unique window into the real-time dynamics of blood flow. In areas of the body as intricate and sensitive as the cerebral vasculature, even slight deviations in flow can be of critical significance [16]. The ability of the db meter to capture these changes, albeit indirectly through sound measurement, could herald a transformative shift in how surgeons approach, evaluate, and conclude their interventions [17].

Beyond its immediate application, the lack of established literature on the db meter in neurosurgery means that there is a vast unexplored territory of potential research topics [16]. These could range from the tool’s precision, sensitivity, and reliability in various surgical contexts, to its potential applications beyond neurosurgery, and even the development of new techniques and protocols centered around its use [16,17]. Without the weight of peer-reviewed publications, the widespread adoption of the dB meter in clinical settings might be met with skepticism. Moreover, the medical community operates on the principle of evidence-based practice; hence, any new tool or technique requires robust evidence to support its use [16,17]. In essence, the dB meter’s relative novelty in neurosurgery is both an exciting frontier and a call to action. Although the device’s potential is palpable, it is up to the medical community to explore, validate, and document its capacities. The journey toward this might be challenging, but the rewards, in terms of improved surgical outcomes and patient safety, promise to be well worth the effort [17]. It is important to note that the use of a DB meter during the clipping procedure may have an impact on the surgical procedure itself. This potential impact should be evaluated by reviewing surgical notes and recordings to determine if any complications or delays occurred due to the use of the dB meter [17].

There are several limitations to consider when interpreting the results of this study such as the sample size used in this study may not be representative of the larger population. The study only focused on the use of a dB meter to measure sound levels during the clipping procedure and did not assess the impact of other factors on sound levels or the effectiveness of other methods for evaluating clipping efficiency. Future studies should consider examining the impact of other factors such as surgical technique, aneurysm location, and patient characteristics on sound levels during aneurysm clipping procedures. Moreover, the study did not include a control group, which limits the ability to conclude the effectiveness of aneurysm clipping in reducing sound levels and exposure dose.

## Conclusions

The results of this study indicate the potential for using sound measurements as a reliable indicator of adequate aneurysm occlusion during clipping procedures. The decibel measurements obtained through the use of a decibel meter in the study can provide valuable information about the sound levels generated during the clipping procedure in cerebral aneurysm surgery. While sound levels can indirectly indicate certain aspects of the surgical procedure, such as the presence or absence of blood flow in the clipped complex, it is important to note that decibel measurements alone may not be sufficient to determine the accuracy or correctness of the clipping itself. In the study, the decibel meter was used in combination with intraoperative MD ultrasonography to assess clipping efficiency. The MD ultrasonography provides direct information about blood flow in the arteries and aneurysm dome, which is a critical factor in determining the success of the clipping procedure.

It is crucial to consider that the decibel meter measurements alone cannot solely determine the correctness of the clipping procedure. The combined use of decibel measurements and MD ultrasonography provides a more comprehensive approach to evaluating the efficacy of the clipping and its impact on blood flow. Clinical expertise and the assessment of the overall surgical outcome remain crucial in determining the success of the procedure. However, further research is needed to confirm these findings and explore the use of sound measurements in other surgical procedures. Furthermore, collaboration between surgical and audiology professionals could lead to the development of new technologies and techniques for the measurement and analysis of sound in surgical settings and could ultimately improve patient outcomes.
